# Closure Device-Related Thrombosis After Anticoagulation With Dabigatran in Patients Undergoing Percutaneous Left Atrial Appendage Closure: Case Reports and Literature Review

**DOI:** 10.3389/fphar.2020.563920

**Published:** 2020-09-08

**Authors:** Xiaoye Li, Qinchun Jin, Xiaochun Zhang

**Affiliations:** ^1^ Department of Pharmacy, Zhongshan Hospital, Fudan University, Shanghai, China; ^2^ Department of Cardiology, Zhongshan Hospital, Fudan University, Shanghai, China

**Keywords:** atrial fibrillation, left atrial appendage closure, dabigatran, device-related thrombosis, novel oral anticoagulation

## Abstract

Percutaneous left atrial appendage closure (LAAC) is an effective and safe operation strategy for stroke prevention in patients who are diagnosed with atrial fibrillation (AF) but cannot tolerate long term anticoagulation medication. We presented four rare cases of thrombosis formation on the occluder device. After the LAAC operation was successfully performed on patients, they followed a course of anticoagulation with dabigatran (110 mg b.i.d.), and device-related thrombosis (DRT) occurred as indicated by a transesophageal echocardiogram (TEE) during the follow-up period. Regressions were achieved after replacing dabigatran with rivaroxaban or warfarin for more than 1 month. No thrombosis or bleeding-related complications occurred in subsequent follow-ups.

## Introduction

Recently, percutaneous left atrial appendage closure (LAAC) has been developed as an effective and safe operation strategy for stroke prevention in patients who are diagnosed with atrial fibrillation (AF) but cannot tolerate long term anticoagulation (Glikson et al., 2019; Holmes et al., 2019; Skurk and Landmesser, 2020). The left atrium plays an important role in the formation of thrombosis for patients with AF, and about 90% of identified cases of left atrium thrombosis are located in the left atrial appendage (LAA) (Kirchhof et al., 2016; Saw et al., 2019). LAAC raises an intriguing concept mainly due to the combination of the reduction in thromboembolism and bleeding risks based on its technical success without the need for long-term pharmacological treatment (Hobohm et al., 2019; Masjuan et al., 2019; Wintgens et al., 2019).

Similar to other implanted devices in the human body, there is a requisite time for full endothelialization on occluders that are exposed to circulating blood (Cornelissen and Vogt, 2019). However, there was significant interindividual variability on anticoagulation before complete endothelialization on devices, which might add to uncertainty on the duration of antithrombotic therapy during this vulnerable time for device-related thrombosis (DRT) (Massarenti and Yilmaz, 2012; Tang et al., 2017). Previous research reported that the incidence of DRT with oral anticoagulants (OAC) was lower than that with antiplatelet therapy (3.1 vs. 1.4%; p = 0.018) (Bergmann et al., 2017). However, the optimal anticoagulation regimen is uncertain owing to the lack of comparative clinical studies on different antithrombotic agents (mainly dabigatran and rivaroxaban). Previous studies have provided definitive evidence on the safety and efficacy of rivaroxaban for post anticoagulation of LAAC (Panikker et al., 2016), but little is known about the anticoagulation effects of dabigatran on LAAC. Current evidence shows that medication with dabigatran fails to prevent thromboembolic complications for patients after stents were implanted and mechanical heart valves replaced (Jaffer et al., 2015).

Given the recent increase in the prescription of novel oral anticoagulants (NOAC), such as dabigatran, for post-implantation anticoagulation after LAAC operations, it seems important to obtain a better understanding of the pharmacology and adverse effects during anticoagulation. Here, we report on four cases of occluder-related thrombosis anticoagulated with dabigatran in patients undergoing LAAC operations.

## Case Studies

**Figure 1:**
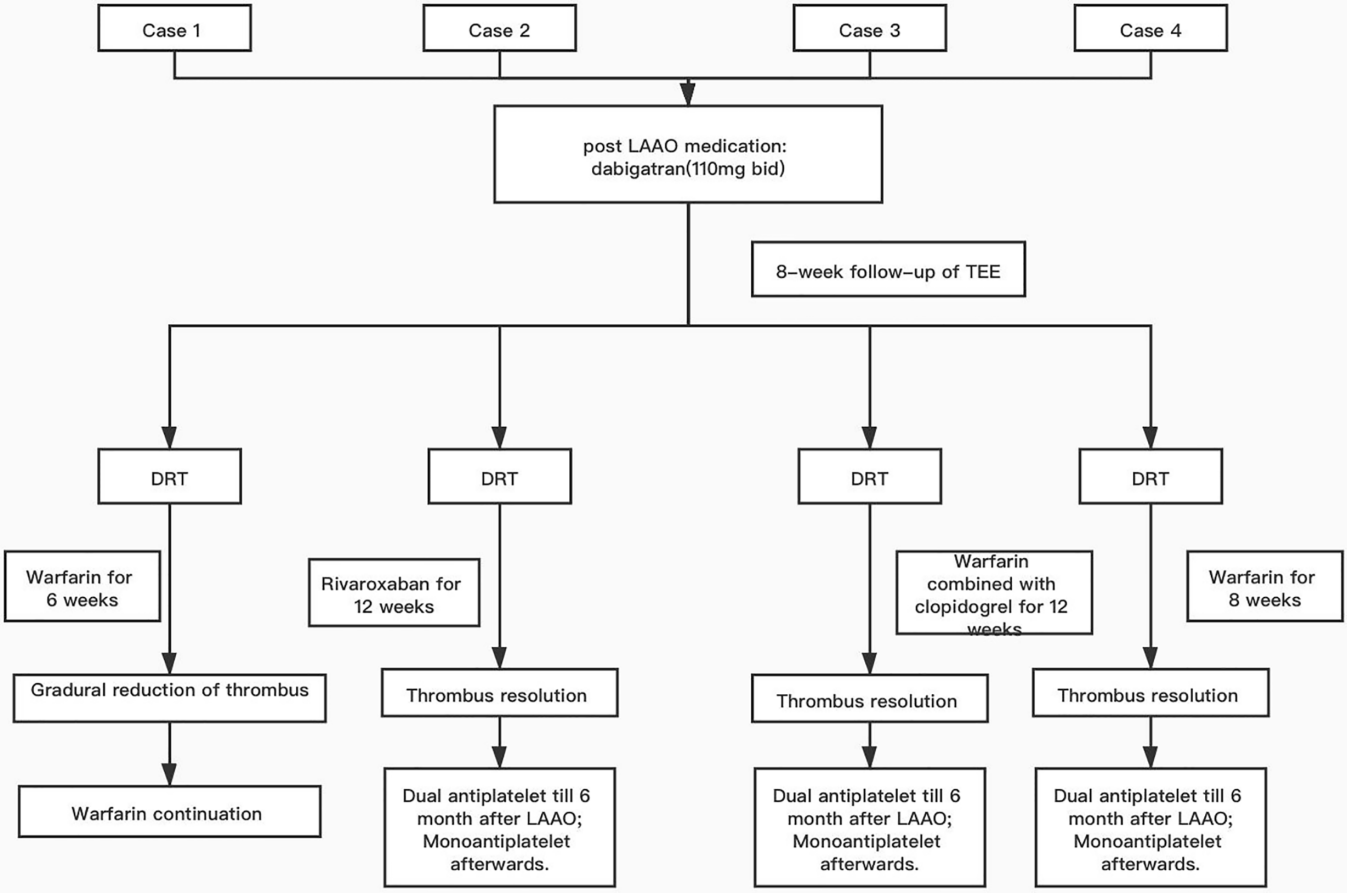
Flow chart of the care process for the presented cases. LAAO, left atrial appendage occlusion; TEE, transesophageal echocardiogram; DRT, device-related thrombosis; dual antiplatelet, 100 mg of aspirin combined with 75 mg of clopidogrel; mono antiplatelet, 100 mg of aspirin only.

**Figure 1** presents progression of clinical picture in our patients.

### Case 1

**Figure 2:**
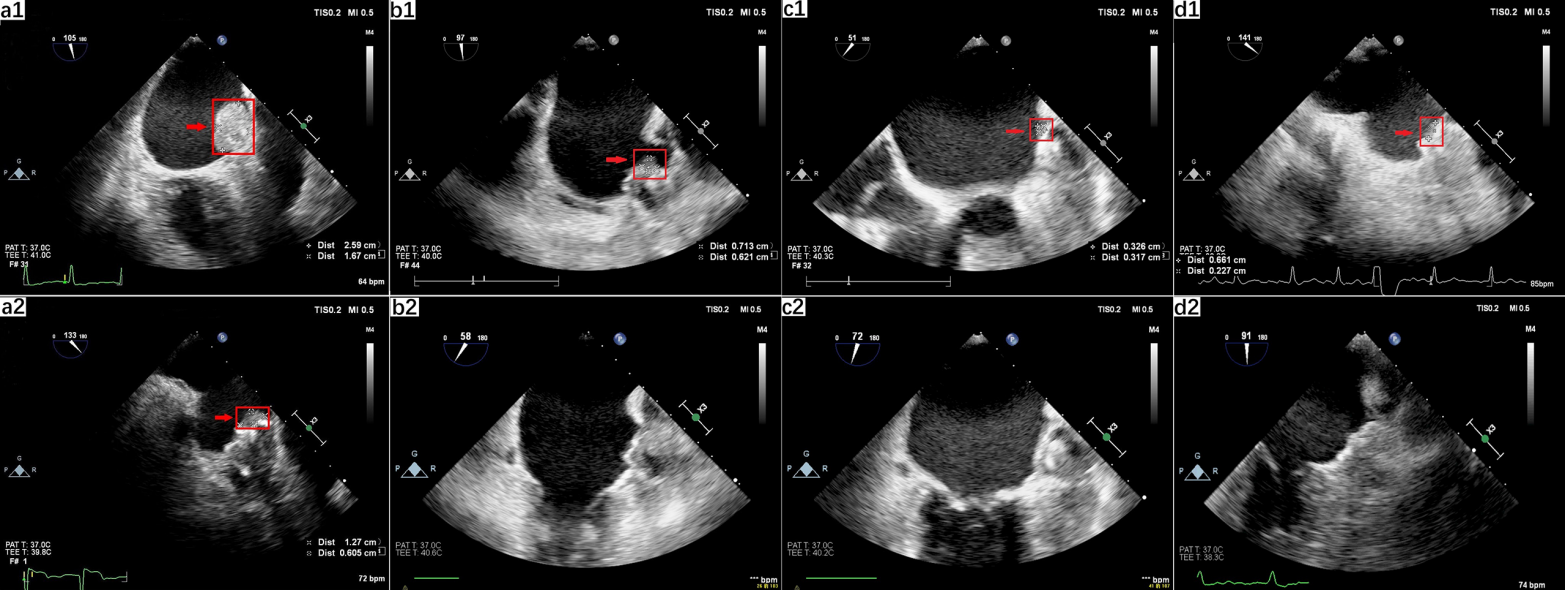
Left atrial appendage occlusion-related thrombus anticoagulated with dabigatran. (a) TEE 2-dimensional view displayed thrombosis (2.59 cm × 1.67 cm) on the surface of the occluder (a1). After six weeks of warfarin treatment, a regression of the thrombosis was shown (1.27 cm × 0.605 cm), which confirmed the diagnosis of device-related thrombosis (a2). (b) TEE revealed a thrombus (0.713 cm × 0.621 cm) on the anterolateral surface of the occluder (b1) and the total disappearance of the thrombus after initiation of anticoagulation therapy with rivaroxaban (b2). (c) TEE check-up revealed abnormal hemodynamics in the LAA, identified as thrombosis (0.713 cm × 0.621 cm) (c1), and total elimination of DRT after 3 months following anticoagulation treatment with warfarin (c2). (d) TEE revealed a thrombus (0.661 cm×0.227 cm) on the surface of the occluder (d1) and the total disappearance of the thrombus after the initiation of anticoagulation therapy with warfarin (d2). Red arrows indicate the thrombosis.

A 78-year-old woman (weight, 73 kg; height, 158 cm; BMI, 29.2 kg/m^2^) developed paroxysmal AF with clinical presentation as palpitations after exercise accompanied by chest discomfort. The electrocardiogram (ECG) showed the typical pattern of AF: irregular RR intervals and no discernible, distinct P waves. She presented a history of hypertension for 10 years mediated with valsartan and amlodipine to control blood pressure. As her CHA_2_DS_2_-VASc score was 4 (female, elderly, and hypertensive), she now had a clear reason for anticoagulation and was temporarily treated with a vitamin K antagonist (VKA) within the therapeutic range [international normalized ratio (INR), 2.0–3.0). One year after warfarin initiation, she was referred to a tertiary cardiology center for further analysis of AF and was considered for the LAAC operation due to her unsuitability for continuous long-term OAC. A 33-mm Watchman occluder device (Boston Scientific, MA, USA) was successfully deployed under general anesthesia in the LAA with a good seal and no leaks. A transesophageal echocardiogram (TEE) detected no mural thrombus. She was discharged with an 8-week course of dabigatran (110 mg b.i.d.) and a follow-up TEE to assess cardiac function and LAAC device positioning, which might affect further anticoagulation strategies. A proton pump inhibitor (PPI; rabeprazole, 10 mg b.i.d.) was prescribed to reduce the risk of gastrointestinal bleeding. A follow-up TEE, performed 6 weeks later, indicated that thrombus had formed on the occluder surface (**Figure 2**, a1). The physicians decided to switch from dabigatran to warfarin (INR, 2.0–3.0) for anticoagulation. Six weeks later, a TEE revealed the gradual reduction of thrombosis formation on the occluder (**Figure 2**, a2), and physicians made an informed decision to continue on warfarin.

### Case 2

A 66−year−old female patient (weight, 65 kg; height, 156 cm; BMI, 26.7 kg/m^2^) visited our clinic for further evaluation after having an ischemic stroke. She reported recurrent palpitations and chest pain for 2 years. The ECG demonstrated typical AF patterns with irregular RR intervals and no discernible, distinct P waves. She had a history of hypertension with currently uncontrolled high blood pressure. Paroxysmal AF was diagnosed, and OAC treatment with warfarin was initiated (the CHA_2_DS_2_-VASc score is 5). After medication with OAC, she suffered upper gastrointestinal bleeding in the following year. She was then transferred to a tertiary cardiology center for a LAAC operation, which provides stroke prevention in patients with nonvalvular AF who are eligible for OAC therapy. As the LAA and left atrium were free of thrombosis, the LAAC operation was performed, and a 30-mm Watchman occluder device (Boston Scientific, MA, USA) was successfully deployed under general anesthesia in the LAA with a good seal and no leaks. She was discharged with an 8-week course of dabigatran (110 mg b.i.d.) for anticoagulation and a follow-up TEE for the assessment of DRT. In consideration of the gastrointestinal bleeding, she was medicated with PPI (rabeprazole, 10 mg b.i.d.). Six weeks after implantation, a TEE revealed thrombosis in the anterolateral surface of the occluder (**Figure 2**, b1). In consideration of warfarin-induced upper gastrointestinal bleeding, the anticoagulation strategy switched from dabigatran to rivaroxaban (15 mg q.d.) due to the appearance of thrombosis, and another TEE was scheduled for 12 weeks later. Under the effective anticoagulation, the new TEE showed a total elimination of thrombosis (**Figure 2**, b2). She suffered no bleeding event after the anticoagulation strategy change in the following 2 years.

### Case 3

A 66-year-old man (weight, 87 kg; height, 170 cm; BMI, 30.1 kg/m^2^) was referred to our cardiovascular center for the management of a recent ischemic stroke complicated by permanent AF despite receiving antiplatelet therapy with gastrointestinal hemorrhage transformation. A 24-h Holter monitor displayed a dominant rhythm as AF. His medical history included hypertension and coronary heart disease treated with nifedipine controlled-release tablets (30 mg q.d.) and clopidogrel (75 mg q.d.). His CHA_2_DS_2_-VASc score was 5, in the high-risk category of thrombosis and his HAS-BLED score was 5, in the category of high bleeding risk. Although there was no strong case against OAC use, neurologists indicated a high risk of cerebral bleeding under anticoagulant therapy. Upon confirming no thrombosis in the LAA and left atrium, a LAAC operation was successfully performed on the patient using a 33-mm Watchman occluder device (Boston Scientific, MA, USA) that was placed accurately on the LAA with no immediate complications. The patient was discharged, and anticoagulated with dabigatran (110 mg b.i.d.) for 8 weeks was combined with PPI (rabeprazole, 10 mg b.i.d.). Two months later, a TEE check-up revealed abnormal hemodynamics in the LAA, suspected as thrombosis (**Figure 2**, c1). The antithrombotic regimen was switched from dabigatran to warfarin (3.75 mg q.d.) within the therapeutic range (INR, 2.0–3.0), combined with clopidogrel (75 mg q.d.). A further TEE was performed to confirm the resolution of DRT after 3 months following anticoagulation treatment (**Figure 2**, c2). Warfarin was stopped, and the patient resumed dual antiplatelet therapy for six more months followed by life-long aspirin. No transient ischemic attacks (TIA) or bleeding related complications occurred in the follow-up examinations.

### Case 4

A 78-year-old man (weight, 78 kg; height, 173 cm; BMI, 26.0 kg/m^2^) suffered a transient ischemic attack (TIA) with clinical presentation as hemiplegia and slurred speech for 5 months. The 24-h Holter monitor displayed paroxysmal AF. The patient had an extensive medical history, including coronary artery disease (CAD), arteriosclerosis obliterans, hypertension, and chronic kidney disease (CKD III). The estimated glomerular filtration rate (eGFR) was calculated as 45 by the modified MDRD equation and was diagnosed as stage III renal dysfunction. He received dabigatran (110 mg b.i.d.) for stroke prevention, and routine urine tests revealed hematuria after anticoagulation initiation. His CHA_2_DS_2_-VASc score was 6 (hypertensive, aged 75 years, and suffering from transient ischemic attacks and vascular disease), and his HAS-BLED score was 6 (hypertension, abnormal renal function, stroke, bleeding, elderly, and drugs). Due to his unsuitability for long term anticoagulation and the high risk of stroke, he was transferred to the cardiology center for further analysis of AF and consideration of LAAC. A 30-mm Watchman occluder device (Boston Scientific, MA, USA) was successfully deployed under general anesthesia in the LAA with a good seal and no leaks. It was confirmed by a TEE that there was no thrombosis in the LAA. He was discharged with an eight-week course of dabigatran (110 mg b.i.d.) for post-implantation anticoagulation treatment, followed by dual antiplatelet therapy (aspirin, 100 mg q.d.; clopidogrel, 75 mg q.d.) for 6 months and aspirin (100 mg q.d.) for life. The routine follow-up TEE for the LAAC device position after 2 months revealed thrombus on the surface of the occluder (**Figure 2**, d1). He was switched from dabigatran to warfarin with a target INR of 2.0–3.0 for prolonged anticoagulation, followed by a repeat TEE scheduled for 8 weeks later. TEE check-ups later revealed the total disappearance of the abnormal thrombosis with the final diagnosis refined as DRT (**Figure 2**, d2). No TIA and bleeding-related complications occurred. Warfarin was stopped and dual antiplatelet therapy (100 mg of aspirin and 75 mg clopidogrel) was initiated instead.

## Discussion

To the best of our knowledge, this is the first report to investigate thrombosis formed on LAA devices associated with dabigatran exposure in patients undergoing percutaneous LAAC. There was a total disappearance of the abnormal thrombosis under anticoagulation conversion from dabigatran to rivaroxaban and warfarin. Our findings indicate that dabigatran is less effective than warfarin and rivaroxaban in reducing thrombosis after LAAC procedures. Our report indicates that postoperative DRT may still take place despite the use of dabigatran.

### Antithrombotic Therapy for Postoperative Care

Previous animal test results showed that device endothelialization might occur over the LAA surface and extend over the adjacent endothelium following occluder implantation (Bass, 2010; Schwartz et al., 2010). In our review, the occurrence of thrombosis on the novel Watchman device is thought to be more frequent in the first few weeks after implantation and to decline with complete endothelialization of the device surface. According to current guidelines, it is recommended that an intensive course of anticoagulation with NOAC is given to patients ineligible for warfarin to facilitate device endothelialization, followed by dual antiplatelet therapy for six months and then lifelong aspirin (Glikson et al., 2019).

### Risk Factors of Thrombosis Formation After LAAC Operation

In our study of the four patients with DRT, routine follow-up TEE identified post-procedure thrombosis formation. Given the common use of LAAC operations in patients with intolerant bleeding risk under OAC, DRT leaves both patients and physicians with a dilemma: it provides an iatrogenic indication for therapeutic anticoagulant therapy and additional TEE check-ups. Many risk factors might contribute to DRT including procoagulant patient-specific factors with a high risk of thrombosis, device implantation specific factors, and inappropriate anticoagulation (Kaneko et al., 2017). Notably, incomplete closures with peri-device leakage were associated with thromboembolic events (Kanderian et al., 2008). It was likely that residual flow around the device into a stagnant LAA pouch might contribute to turbulent blood flow and enhance platelet adhesion and clot formation (Saw et al., 2017). In our four cases, TEE imaging displayed a good seal and no leaks for occluders inserted on the LAA which ruled out device implantation specific factors. The mechanism of thrombosis formation after LAAC operations was more likely involved with high-risk procoagulant features and inappropriate anticoagulation. Also, the deployed device size may be another probable contributor to DRT. A previous study reported that the device size was larger in patients with development of thrombus (29.3 ± 3.8 mm vs. 25.7 ± 3.2 mm, respectively) after the Watchman device implantation (Kubo et al., 2017). The increased risk of thrombus formation may be explained by the larger area of the fabric on the larger device. In our cases, the patients were implanted with a device size of 33 mm, which could add up to the risk of postoperative DRT. However, we think that anticoagulation with dabigatran played a principal role, since complete thrombus resolution was observed after switching to alternative anticoagulation therapy.

### Post-LAAC Anticoagulation With NOAC for DRT Prevention

After successful Watchman implantation, a post-thrombotic regimen with NOAC is considered as a substitute for patients who are unable to tolerate a short duration of warfarin until complete endothelialization of LAA devices (Reddy et al., 2017). Effective and safe therapy with warfarin requires continuous monitoring of prothrombin time (PT) and INR levels to adjust the dose of warfarin (Numao et al., 2017). Warfarin has a narrow therapeutic window with an INR in the range of 2.0–3.0, and many factors can influence the warfarin dosing algorithm including patient characteristics such as body mass index (BMI), age, comorbidities, concomitant drugs, and diet, as well as genetic variants for warfarin metabolism *via* cytochrome P450 (CYP) 2C9 and genetic differences in recycling vitamin K through vitamin K epoxide reductase (VKORC1) (Verhoef et al., 2014; Gage et al., 2017; Drozda et al., 2018). Many randomized clinical trials (RCT) demonstrated that NOAC was superior to warfarin in efficacy and safety and NOACs seemed to be more effective and safer for short period anticoagulation compared with warfarin for patients post LAAC operation (Connolly et al., 2009; Reddy et al., 2017). However, there are currently no comparisons of clinical efficacy and safety with NOACs for patients undergoing LAAC operations.

### Thrombosis on Closure Devices Anticoagulated With Dabigatran

Dabigatran, a direct inhibitor of thrombin, has been shown to be an alternative anticoagulant for patients intolerant of warfarin in the prevention and treatment of thromboembolic disease (Connolly et al., 2009). In these four cases, the follow-up TEE imaging displayed thrombosis with DRT; therefore, anticoagulant adjustment was needed to treat the thrombus. At present, there is no relevant literature about the mechanism for dabigatran medication increasing thrombosis risks post LAAC operation. One clinical study demonstrated that dabigatran medication could increase platelet reactivity by enhancing thrombin receptor density on thrombocytes, contributing to increased risk of myocardial infarction (Franchi et al., 2016). Dabigatran-enhanced platelet reactivity induced by the thrombin receptor activating peptide is specific to thrombin-induced platelet activation (Reilly et al., 2014; Yau et al., 2014). This might be one reason for the occurrence of DRT after a LAAC operation.

Like other blood-contacting medical devices, the occluder components trigger thrombosis formation *via* activation of the intrinsic pathway. It is possible that after LAAC higher than the conventionally used doses of dabigatran (i.e., 100 mg b.i.d.) may be required to prevent DRT. Also, some studies indicate that standard dosing regimens may be associated with lower dabigatran plasma concentrations in obese patients because of higher volumes of distribution (Yau et al., 2014). BMI of >25 was the cut-off point according to the World Health Organization for obesity (Haschke et al., 2016). This might lead to a reduction in the anti-thrombosis effect of dabigatran and an increase in the incidence of DRT (Lucijanic et al., 2020). One probable explanation for our four cases with closure device-related thrombosis anticoagulated with dabigatran was the suboptimal drug dosage levels which potentially increased the risk of thrombosis.

Another probable mechanism for attenuated anticoagulation might be a dabigatran/PPI interaction leading to decreased dabigatran plasma concentration. The bioavailability of dabigatran etexilate is pH-dependent, and co-administration with PPI could increase gastric pH levels, which might decrease the dissolution of dabigatran etexilate (Schnierer et al., 2020). Three cases in our study were in concomitant medication with PPI after the LAAC operation.

The common genetic variants of CES1 and ABCB1 have been identified to potentially account for the interindividual variations in dabigatran plasma levels which could lead to varied anticoagulation therapeutic responses (Sychev et al., 2020). It has been proven that the single nucleotide polymorphism (SNP) in the CES1 gene (rs2244613) could alter dabigatran metabolism, leading to lower trough concentrations and increasing thrombosis risks (Sychev et al., 2020). However, we did not investigate the two associated genetic variants in these four cases.

### Thrombus Reversal After Transferring to Rivaroxaban and Warfarin

After the confirmation of thrombus, we switched to rivaroxaban and warfarin for anticoagulation, leading to a gradual reduction of thrombosis. In contrast to the anticoagulation mechanism of dabigatran, rivaroxaban is a factor X inhibitor and selectively inhibits FXa with a rapid onset of action, which was beneficial to the prevention of thrombosis and platelet aggregation (Enomoto et al., 2017). Many clinical trials demonstrated that oral rivaroxaban co-administration with antiplatelet therapy could decrease the incidence of thromboembolism with thromboembolic events and deaths due to cardiovascular events, myocardial infarction and stroke (Rubboli et al., 2015).

## Conclusion

DRT after LAAO has been acknowledged to have a strong correlation with the risks of postoperative stroke and systematic embolic events. Hence, in these cases, the patients are still confronted with neurologic morbidity and mortality. Moreover, the incidence of re-hospitalization and outpatient follow-up visits for adjusting anticoagulation medication might also lead to a waste of medical resources and the added burden of medical expenses. Besides procedural factors including larger device size and deep implantation, which were related to the risk of thrombus formation, novel oral anticoagulant usage such as dabigatran at discharge was also associated with a risk of thrombus formation. The patient BMI and co-administration with PPI should be taken into account during the medication process. The management of such a complication is not standardized, and transferring to rivaroxaban and warfarin might be an alternative anticoagulation strategy. The limitation was the absence of dabigatran related genetic testing for these patients.

## Data Availability Statement

All datasets presented in this study are included in the article/supplementary material.

## Ethics Statement

Written informed consent was obtained from the individuals for the publication of any potentially identifiable images or data included in this article.

## Author Contributions

XL wrote manuscript and performed literature search and review. XZ provided case and corrected manuscript. QJ provided case and corrected manuscript.

## Conflict of Interest

The authors declare that the research was conducted in the absence of any commercial or financial relationships that could be construed as a potential conflict of interest.
